# Data on the domestic processed output, balancing items, and solid waste potential for five major world economies

**DOI:** 10.1016/j.dib.2018.12.072

**Published:** 2018-12-26

**Authors:** Heinz Schandl, Alessio Miatto

**Affiliations:** a Commonwealth Scientific and Industrial Research Organisation (CSIRO), Canberra, Australia; b Fenner School of Environment and Society, Australian National University (ANU), Canberra, Australia; c Graduate School of Environmental Studies, Nagoya University, Nagoya, Japan; d School of Forestry & Environmental Studies, Yale University, New Haven, CT, United States

**Keywords:** Domestic processed output (DPO), Material flow analysis, Balancing items, Solid waste potential

## Abstract

This data article reports the domestic processed output (DPO), balancing items, and solid waste potential for five major world economies (Australia, China, Germany, Japan, and the United States of America) for the years 1990–2015. The main DPO database assembles data from a number of national and international sources. Linking this with data on domestic material consumption and the calculation of balancing items, we have been able to provide fully balanced inputs and outputs for these five economies. To integrate poor statistics on solid waste, we modelled an additional solid waste potential account based on a stock and flow driven model that estimates solid waste output for the year 2015. These data underlies the research published in “On the importance of linking inputs and outputs in material flow accounts. The Weight of Nations report revisited” (Schandl and Miatto, 2018).

**Specifications table**

**Table:** 

Subject area	*Environmental sciences*
More specific subject area	*Ecology*
Type of data	*Tables and figures*
How data were acquired	*Collection of statistical data (for DPO account), own calculations (for balancing items), and modelled (for solid waste potential)*
Data format	*Raw and analysed*
Experimental factors	*Data are sourced from major international and, when unavailable, national agencies for five countries: Australia, China, Germany, Japan, and the United States of America*
Experimental features	*The DPO account is comprised of untreated data from several sources; the balancing items are calculated in accordance with the economy-wide material flow account manual issued by Eurostat; the solid waste potential is estimated through a depreciation model fed by material extraction and trade statistics issued by the International Resource Panel.*
Data source location	*International sources (FAO, IEA, UNFCCC), national statistical accounts.*
Data accessibility	*Data are included in this article*
Related research article	*Schandl, Heinz, and Alessio Miatto. “On the Importance of Linking Inputs and Outputs in Material Flow Accounts. The Weight of Nations Report Revisited.” Journal of Cleaner Production*[1].

**Value of the data**

• This data article provides a complete dataset on the material outflows of five major world economies. For the first time we can compare the emissions and waste of developed old world countries (Germany and Japan), developed new world countries (Australia and the United States), and developing Asian countries (China) on a per-capita level.
• This dataset can be integrated and expanded with other countries in future and offers a valid benchmarking point for monitoring emissions and waste production on a per-capita level, linking to the sustainability efforts outlined by the sustainable development goals (SDGs).
• The calculation of the balancing items makes explicit the quantification of the net additions to stock (NAS), which can be compared to empirical calculations to assess the soundness of stock accumulation models.
• Disaggregated data on material types reported in the solid waste potential permits the evaluation of the quality of reported solid waste production, offering a clear indication of which sectors are the main contributors to these categories, and links to ongoing discourse on the circularity of the economy.

## 1 Data

The composition of the DPO data was made by collecting the comprising elements from a number of national and international sources [2], [3], [4], [5], [6], [7]. These data did not undergo any further processing, since our intention was to assess the availability of data and countries’ capabilities to compile DPO data without any modelling or interpolation.

Data on balancing items, both on the input and output sides have been calculated using the economy-wide material flow analysis manual issued by the European Statistical Office Eurostat [8], [9]. The data on material inflows, trade, domestic material consumption (DMC), domestic processed output (DPO), balancing items, and net additions to stock (NAS) are reported in Table 1, Table 2, Table 3, Table 4, Table 5. Note that these are all yearly flows, and do not refer to a cumulative total.

**Table 1:** Australia׳s material balance from 1990 to 2015. Units in thousand tonnes.

**Australia׳s material balance**								
**Unit: thousand tonnes**								
**Year**	**Domestic extraction (DE)**	**Imports**	**Exports**	**Domestic material consumption (DMC)**	**Domestic processed output (DPO)**	**Balancing items: input side**	**Balancing items: output side**	**Net additions to stock (NAS)**
*1990*	908,514	24,965	265,288	668,191	401,243	502,314	369,569	399,694
*1991*	919,444	25,770	302,911	642,303	401,373	482,646	361,016	362,560
*1992*	943,094	26,572	299,479	670,187	403,206	453,009	363,107	356,884
*1993*	985,525	30,955	319,345	697,135	407,209	445,587	363,196	372,317
*1994*	985,355	33,807	333,434	685,728	415,240	450,084	365,735	354,837
*1995*	1,042,701	35,128	347,906	729,924	426,217	450,097	377,575	376,228
*1996*	1,118,937	36,331	363,548	791,720	436,643	462,090	385,196	431,971
*1997*	1,161,494	40,650	409,250	792,893	448,616	470,350	383,690	430,937
*1998*	1,218,310	39,477	408,017	849,770	465,243	479,436	393,611	470,352
*1999*	1,227,227	43,059	417,319	852,967	477,390	495,646	390,967	480,256
*2000*	1,284,431	43,301	459,293	868,439	487,038	506,722	400,384	487,739
*2001*	1,313,474	44,098	469,505	888,067	495,290	524,277	403,470	513,583
*2002*	1,291,501	46,817	491,035	847,282	500,590	524,419	395,020	476,091
*2003*	1,375,504	47,777	512,591	910,690	508,074	520,245	402,245	520,615
*2004*	1,394,463	49,091	558,561	884,994	519,520	526,654	397,364	494,763
*2005*	1,461,576	53,505	600,701	914,379	525,837	544,543	409,665	523,420
*2006*	1,426,286	55,408	614,774	866,921	533,065	562,488	406,442	489,902
*2007*	1,502,948	59,210	641,246	920,912	543,129	565,601	417,443	525,940
*2008*	1,526,834	65,757	691,402	901,190	545,714	554,465	413,700	496,241
*2009*	1,624,230	59,885	763,038	921,078	551,413	554,846	415,003	509,508
*2010*	1,673,832	67,425	841,706	899,551	548,500	535,374	404,711	481,714
*2011*	1,728,499	70,915	881,751	917,663	548,991	532,156	417,271	483,557
*2012*	1,804,101	72,159	965,826	910,434	552,880	520,362	417,612	460,304
*2013*	1,936,692	75,016	1,091,410	920,298	546,273	515,196	421,657	467,564
*2014*	2,108,788	75,456	1,275,297	908,947	543,522	514,879	420,167	460,136
*2015*	2,172,404	76,979	1,333,299	916,083	551,911	512,174	410,731	465,615

**Table 2:** China׳s material balance from 1990 to 2015. Units in thousand tonnes.

**China׳s material balance**								
**Unit: thousand tonnes**								
**Year**	**Domestic extraction (DE)**	**Imports**	**Exports**	**Domestic material consumption (DMC)**	**Domestic processed output (DPO)**	**Balancing items: input side**	**Balancing items: output side**	**Net additions to stock (NAS)**
*1990*	6,524,453	52,832	91,928	6,485,356	3,908,493	3,527,557	2,882,293	3,222,127
*1991*	6,912,487	61,474	109,070	6,864,891	4,078,970	3,651,385	2,948,701	3,488,605
*1992*	7,910,739	95,516	114,929	7,891,326	4,263,069	3,769,797	3,010,803	4,387,251
*1993*	8,737,623	139,976	121,367	8,756,232	4,677,613	4,079,004	3,120,036	5,037,587
*1994*	9,481,551	164,389	118,093	9,527,847	4,902,050	4,226,413	3,213,164	5,639,047
*1995*	10,347,813	151,788	131,828	10,367,772	5,333,238	4,537,552	3,426,016	6,146,070
*1996*	10,669,359	163,270	140,420	10,692,209	5,423,323	4,651,750	3,593,729	6,326,907
*1997*	10,856,731	195,873	161,480	10,891,123	5,428,848	4,577,431	3,401,858	6,637,848
*1998*	11,173,518	186,806	143,299	11,217,026	5,666,734	4,672,957	3,506,249	6,717,001
*1999*	11,430,100	208,469	136,169	11,502,400	5,594,807	4,649,585	3,569,640	6,987,538
*2000*	11,716,680	280,433	191,314	11,805,799	5,871,066	4,887,334	3,685,401	7,136,666
*2001*	12,316,931	320,789	228,598	12,409,122	6,114,674	4,997,365	3,704,874	7,586,939
*2002*	13,008,985	370,636	257,522	13,122,100	6,484,786	5,248,923	3,761,182	8,125,054
*2003*	14,248,104	474,637	265,679	14,457,063	7,289,882	5,831,972	3,872,770	9,126,382
*2004*	15,611,568	602,660	259,883	15,954,345	8,251,396	6,536,407	4,103,249	10,136,106
*2005*	16,641,788	682,334	293,720	17,030,402	9,162,435	7,165,116	4,225,812	10,807,271
*2006*	18,176,847	773,943	342,086	18,608,704	9,993,790	7,743,290	4,359,617	11,998,588
*2007*	19,550,432	895,369	350,890	20,094,911	10,818,062	8,239,974	4,467,896	13,048,927
*2008*	20,275,300	968,194	314,666	20,928,827	11,149,039	8,451,769	4,637,932	13,593,626
*2009*	22,379,811	1,329,048	222,131	23,486,728	11,721,226	8,880,051	4,817,626	15,827,926
*2010*	25,009,193	1,439,157	265,405	26,182,945	12,628,582	9,431,881	5,081,277	17,904,967
*2011*	27,016,123	1,631,510	281,534	28,366,100	14,326,870	10,189,513	5,285,505	18,943,238
*2012*	28,003,974	1,806,196	272,038	29,538,132	14,640,386	10,446,270	5,421,840	19,922,176
*2013*	29,151,179	1,999,941	301,968	30,849,152	15,267,004	10,984,911	5,536,623	21,030,437
*2014*	29,922,955	2,056,170	380,270	31,598,855	15,804,465	11,375,784	5,539,526	21,630,648
*2015*	30,844,488	2,076,598	381,564	32,539,522	16,377,547	11,768,025	5,558,737	22,371,263

**Table 3:** Germany׳s material balance from 1990 to 2015. Units in thousand tonnes.

**Germany׳s material balance**								
**Unit: thousand tonnes**								
**Year**	**Domestic extraction (DE)**	**Imports**	**Exports**	**Domestic material consumption (DMC)**	**Domestic processed output (DPO)**	**Balancing items: input side**	**Balancing items: output side**	**Net additions to stock (NAS)**
*1990*	1,143,971	242,122	47,011	1,339,082	1,466,596	1,178,076	675,284	375,278
*1991*	1,028,241	371,202	127,550	1,271,893	1,439,805	1,136,918	646,951	322,055
*1992*	1,019,622	393,957	133,581	1,279,998	1,400,454	1,088,718	619,155	349,107
*1993*	984,153	371,655	120,720	1,235,088	1,381,505	1,074,317	607,962	319,938
*1994*	1,023,239	399,206	138,149	1,284,296	1,379,202	1,057,034	600,089	362,039
*1995*	954,031	399,604	134,873	1,218,762	1,379,933	1,056,757	602,451	293,134
*1996*	911,283	402,296	139,221	1,174,357	1,400,215	1,086,156	620,226	240,073
*1997*	935,199	410,832	147,310	1,198,721	1,382,060	1,059,152	614,305	261,507
*1998*	1,027,037	430,088	159,855	1,297,270	1,375,765	1,048,281	606,244	363,542
*1999*	1,079,484	415,066	165,343	1,329,206	1,358,337	1,015,999	593,000	393,867
*2000*	1,045,966	438,118	177,418	1,306,666	1,367,027	1,014,324	588,999	364,963
*2001*	990,187	436,633	180,085	1,246,734	1,384,343	1,034,976	600,044	297,323
*2002*	954,943	430,307	188,733	1,196,518	1,363,542	1,042,450	591,496	283,929
*2003*	931,073	423,322	185,859	1,168,536	1,355,519	1,041,166	585,292	268,892
*2004*	951,441	448,215	204,207	1,195,449	1,338,828	1,029,144	594,692	291,074
*2005*	906,920	457,087	227,019	1,136,987	1,323,209	1,007,679	583,532	237,926
*2006*	932,377	476,080	234,124	1,174,333	1,344,764	1,019,738	584,149	265,158
*2007*	1,017,324	478,134	248,760	1,246,698	1,324,454	981,313	566,913	336,644
*2008*	1,000,703	480,319	244,656	1,236,366	1,330,534	987,105	578,950	313,987
*2009*	975,248	414,392	211,530	1,178,110	1,250,408	925,950	563,781	289,872
*2010*	948,269	459,650	227,706	1,180,212	1,298,042	958,481	556,801	283,850
*2011*	996,155	467,756	233,060	1,230,851	1,297,288	938,670	554,537	317,696
*2012*	966,890	459,045	223,184	1,202,752	1,286,701	943,783	556,733	303,100
*2013*	945,872	474,331	225,055	1,195,148	1,311,023	963,656	562,092	285,689
*2014*	961,901	474,452	229,494	1,206,859	1,282,621	914,521	547,093	291,666
*2015*	960,308	489,052	237,773	1,211,588	1,281,274	919,783	555,290	294,807

**Table 4:** Japan׳s material balance from 1990 to 2015. Units in thousand tonnes.

**Japan׳s material balance**								
**Unit: thousand tonnes**								
**Year**	**Domestic extraction (DE)**	**Imports**	**Exports**	**Domestic material consumption (DMC)**	**Domestic processed output (DPO)**	**Balancing items: input side**	**Balancing items: output side**	**Net additions to stock (NAS)**
*1990*	995,283	661,421	38,541	1,618,164	1,659,749	1,224,391	609,824	572,981
*1991*	1,000,778	670,969	44,055	1,627,692	1,671,447	1,222,587	607,214	571,619
*1992*	966,312	660,334	51,575	1,575,071	1,686,353	1,241,571	624,147	506,142
*1993*	939,752	663,618	59,317	1,544,054	1,671,954	1,231,296	618,177	485,219
*1994*	950,235	696,444	61,746	1,584,933	1,743,731	1,301,825	650,507	492,520
*1995*	935,558	713,707	61,139	1,588,126	1,745,460	1,310,392	648,242	504,816
*1996*	970,064	716,398	56,661	1,629,800	1,769,003	1,324,354	656,294	528,857
*1997*	943,679	731,607	61,976	1,613,310	1,776,713	1,320,127	656,003	500,721
*1998*	894,996	692,631	61,735	1,525,892	1,733,917	1,281,980	640,327	433,628
*1999*	882,036	709,772	61,356	1,530,452	1,759,533	1,316,654	652,691	434,882
*2000*	888,037	755,793	68,435	1,575,394	1,790,179	1,344,010	668,020	461,205
*2001*	852,529	724,700	73,450	1,503,780	1,763,054	1,314,843	648,213	407,355
*2002*	811,967	736,679	78,674	1,469,972	1,792,832	1,348,719	658,028	367,830
*2003*	786,735	752,947	78,230	1,461,453	1,815,962	1,355,512	669,134	331,868
*2004*	780,965	771,830	84,785	1,468,010	1,821,686	1,357,077	670,143	333,258
*2005*	775,700	770,056	90,888	1,454,868	1,832,355	1,366,113	670,036	318,589
*2006*	794,162	765,053	94,657	1,464,558	1,808,479	1,352,259	665,598	342,740
*2007*	774,526	778,955	98,599	1,454,882	1,841,995	1,387,343	675,449	324,781
*2008*	723,663	764,253	100,896	1,387,020	1,736,090	1,316,796	656,297	311,429
*2009*	664,464	667,282	96,535	1,235,211	1,647,485	1,237,279	619,487	205,518
*2010*	632,834	741,528	107,166	1,267,195	1,694,271	1,292,776	643,055	222,644
*2011*	625,003	737,899	101,051	1,261,851	1,734,574	1,349,813	666,101	210,990
*2012*	606,466	756,986	95,896	1,267,557	1,764,355	1,386,853	682,602	207,452
*2013*	587,835	769,296	106,938	1,250,193	1,786,413	1,398,892	679,839	182,833
*2014*	568,086	751,432	104,184	1,215,333	1,746,087	1,353,343	664,090	158,500
*2015*	547,738	748,320	108,566	1,187,492	1,699,544	1,314,899	650,829	152,018

**Table 5:** United States’ material balance from 1990 to 2015. Units in thousand tonnes.

**United States of America׳s material balance**								
**Unit: thousand tonnes**								
**Year**	**Domestic extraction (DE)**	**Imports**	**Exports**	**Domestic material consumption (DMC)**	**Domestic processed output (DPO)**	**Balancing items: input side**	**Balancing items: output side**	**Net additions to stock (NAS)**
*1990*	6,669,981	586,041	360,758	6,895,265	6,016,539	5,324,402	3,019,788	3,183,340
*1991*	6,381,938	578,327	374,520	6,585,746	5,961,979	5,250,490	2,986,425	2,887,832
*1992*	6,593,860	610,639	357,135	6,847,364	6,082,995	5,376,686	3,057,863	3,083,192
*1993*	6,561,635	670,345	325,941	6,906,039	6,208,654	5,507,707	3,075,207	3,129,885
*1994*	7,053,823	726,983	313,906	7,466,900	6,324,129	5,626,292	3,220,948	3,548,115
*1995*	7,008,314	722,627	376,898	7,354,043	6,391,388	5,684,097	3,170,756	3,475,997
*1996*	7,181,130	777,710	368,312	7,590,528	6,599,768	5,853,642	3,256,498	3,587,904
*1997*	7,476,281	836,166	353,586	7,958,861	6,688,037	5,965,911	3,314,260	3,922,476
*1998*	7,739,691	894,779	348,462	8,286,009	6,740,476	6,036,333	3,351,921	4,229,944
*1999*	7,726,687	915,277	337,238	8,304,725	6,844,872	6,094,716	3,345,613	4,208,956
*2000*	7,755,532	975,268	368,792	8,362,008	7,038,313	6,252,743	3,390,036	4,186,402
*2001*	7,610,081	995,689	352,503	8,253,268	6,934,645	6,258,527	3,450,583	4,126,567
*2002*	7,399,839	984,659	344,655	8,039,843	6,978,861	6,234,015	3,378,332	3,916,665
*2003*	7,391,047	1,031,376	357,523	8,064,901	7,038,613	6,286,176	3,419,930	3,892,534
*2004*	7,778,052	1,120,078	379,543	8,518,588	7,171,775	6,406,989	3,476,422	4,277,381
*2005*	8,003,532	1,159,465	392,538	8,770,459	7,216,860	6,400,437	3,473,417	4,480,619
*2006*	7,860,070	1,178,618	416,881	8,621,807	7,143,762	6,344,062	3,489,785	4,332,321
*2007*	7,499,056	1,123,062	451,273	8,170,845	7,241,283	6,440,498	3,518,183	3,851,877
*2008*	7,100,313	1,060,229	514,461	7,646,082	7,040,747	6,256,749	3,459,657	3,402,427
*2009*	6,381,275	897,903	472,520	6,806,658	6,547,674	5,853,325	3,375,648	2,736,661
*2010*	6,520,897	939,472	545,959	6,914,410	6,749,370	6,001,712	3,383,101	2,783,651
*2011*	6,543,501	921,939	615,773	6,849,667	6,618,925	5,873,489	3,369,085	2,735,145
*2012*	6,444,891	855,903	623,762	6,677,031	6,412,777	5,688,631	3,326,775	2,626,109
*2013*	6,615,207	822,074	622,511	6,814,769	6,557,752	5,785,556	3,337,792	2,704,782
*2014*	6,765,225	755,059	613,142	6,907,143	6,619,337	5,908,434	3,441,391	2,754,849
*2015*	6,667,735	758,952	623,033	6,803,654	6,459,897	5,842,912	3,495,164	2,691,506

**Fig. 1:**
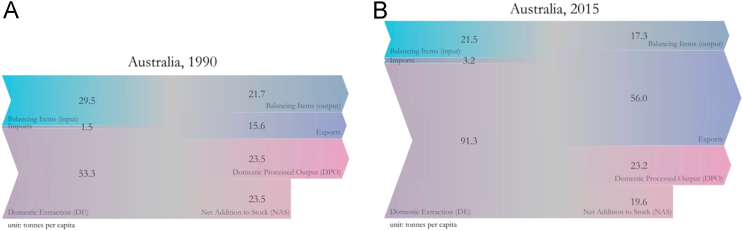
Material balance for Australia. Fig. 1A refers to year 1990, while Fig. 1B refers to year 2015.

**Fig. 2:**
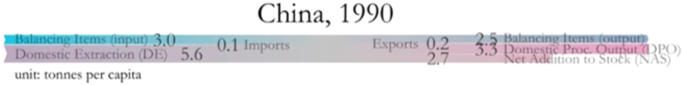
Material balance for China. This figure refers to year 1990, while the figure for year 2015 can be found in Schandl and Miatto [1].

**Fig. 3:**
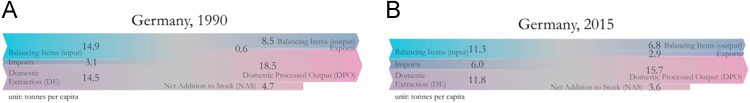
Material balance for Germany. Fig. 3A refers to year 1990, while Fig. 3B refers to year 2015.

**Fig. 4:**
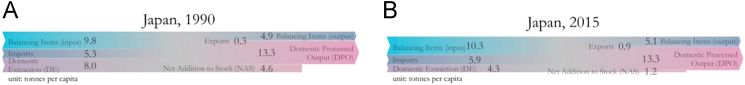
Material balance for Japan. Fig. 4A refers to year 1990, while Fig. 4B refers to year 2015.

**Fig. 5:**
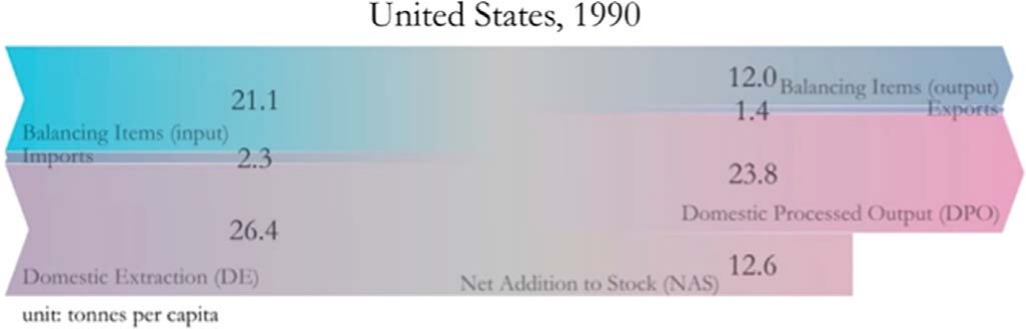
Material balance for the United States of America. This figure refers to year 1990, while the figure for year 2015 can be found in Schandl and Miatto [1].

The results shown above can be visually represented as flows that either enter, exit, or are stocked in each economy. The following figures display a graphical representation of the per-capita material balance for years 1990 and 2015 for the five countries in our case study (Fig. 1, Fig. 2, Fig. 3, Fig. 4, Fig. 5). Note that all the figures are in scale with each other, to better give the reader a sense of the total amount of materials used by the average resident of each country.

Domestic processed output is comprised of five categories: emissions to air, emissions to land, emissions to water, dissipative use of products, and dissipative losses. Emissions to air are gases emitted into the atmosphere such as carbon dioxide (CO_2_), methane (CH_4_), nitrous oxide (N_2_O), et cetera. Note that all these gases are reported in mass terms, and not according to their global warming potential in carbon dioxide equivalent (CO_2_-eq). Emissions to land account for the solid waste reported official statistics on solid waste production. Emissions to water comprise all substances that are released in water. Note that this value considers only substances that are not properly treated in waste water management plants. Dissipative use of products are those materials that are voluntarily used and dispersed in the environment, such as fertilizers, seeds, pesticides, and so on. Dissipative losses refer to products that are unintentionally dispersed into the environment, such as rubber from vehicle tires. The values of the components of the domestic processed output for the countries we analysed are reported in Table 6, Table 7, Table 8, Table 9, Table 10.

**Table 6:** Components of Australia׳s domestic processed output from 1990 to 2015. All units are in thousand tonnes.

**Australia׳s Domestic Processed Output**					
**Unit: thousand tonnes**					
**Year**	**Emissions to air**	**Emissions to land**	**Emissions to water**	**Dissipative use of products**	**Dissipative losses**
*1990*	311,689	32,716	n/a	56,838	n/a
*1991*	312,141	32,859	n/a	56,374	n/a
*1992*	314,713	34,193	n/a	54,299	n/a
*1993*	318,679	35,579	n/a	52,951	n/a
*1994*	324,255	36,962	n/a	54,022	n/a
*1995*	335,503	38,422	n/a	52,292	n/a
*1996*	343,210	39,939	n/a	53,494	n/a
*1997*	352,661	41,712	n/a	54,243	n/a
*1998*	367,154	43,801	n/a	54,288	n/a
*1999*	377,779	45,495	n/a	54,117	n/a
*2000*	385,090	46,373	n/a	55,575	n/a
*2001*	392,458	48,160	n/a	54,673	n/a
*2002*	397,064	49,637	n/a	53,889	n/a
*2003*	404,664	51,695	n/a	51,715	n/a
*2004*	413,149	53,351	n/a	53,020	n/a
*2005*	417,430	54,940	n/a	53,467	n/a
*2006*	423,839	57,000	n/a	52,226	n/a
*2007*	432,925	59,000	n/a	51,204	n/a
*2008*	435,491	61,000	n/a	49,224	n/a
*2009*	441,975	61,000	n/a	48,437	n/a
*2010*	440,304	61,000	n/a	47,196	n/a
*2011*	436,370	62,000	n/a	50,621	n/a
*2012*	438,737	63,000	n/a	51,143	n/a
*2013*	431,024	63,000	n/a	52,249	n/a
*2014*	427,633	64,000	n/a	51,889	n/a
*2015*	434,436	64,000	n/a	53,475	n/a

**Table 7:** Components of China׳s domestic processed output from 1990 to 2015. All units are in thousand tonnes.

**China׳s Domestic Processed Output**					
**Unit: thousand tonnes**					
**Year**	**Emissions to air**	**Emissions to land**	**Emissions to water**	**Dissipative use of products**	**Dissipative losses**
*1990*	3,278,112	372,877	n/a	257,505	n/a
*1991*	3,405,957	407,554	n/a	265,459	n/a
*1992*	3,528,987	465,427	n/a	268,655	n/a
*1993*	3,878,148	530,121	n/a	269,344	n/a
*1994*	4,020,684	599,037	n/a	282,328	n/a
*1995*	4,326,939	708,726	n/a	297,573	n/a
*1996*	4,395,242	721,441	n/a	306,640	n/a
*1997*	4,381,032	736,102	n/a	311,714	n/a
*1998*	4,477,828	884,015	n/a	304,891	n/a
*1999*	4,407,072	874,101	n/a	313,634	n/a
*2000*	4,640,248	910,671	n/a	320,147	n/a
*2001*	4,781,581	991,373	14,048	327,672	n/a
*2002*	5,075,945	1,057,047	13,670	338,124	n/a
*2003*	5,738,392	1,194,823	13,330	343,338	n/a
*2004*	6,512,000	1,365,343	13,392	360,662	n/a
*2005*	7,264,012	1,511,874	14,142	372,408	n/a
*2006*	7,922,034	1,674,663	14,282	382,811	n/a
*2007*	8,496,739	1,919,251	13,818	388,253	n/a
*2008*	8,675,415	2,069,217	13,207	391,200	n/a
*2009*	9,096,639	2,211,069	12,775	400,743	n/a
*2010*	9,621,514	2,583,358	12,381	411,329	n/a
*2011*	10,473,441	3,425,988	30,023	397,418	n/a
*2012*	10,713,293	3,495,904	29,240	401,950	n/a
*2013*	11,351,828	3,480,974	28,496	405,706	n/a
*2014*	11,897,083	3,471,137	28,042	408,202	n/a
*2015*	12,442,339	3,501,970	27,395	405,843	n/a

**Table 8:** Components of Germany׳s domestic processed output from 1990 to 2015. All units are in thousand tonnes.

**Germany׳s Domestic Processed Output**					
**Unit: thousand tonnes**					
**Year**	**Emissions to air**	**Emissions to land**	**Emissions to water**	**Dissipative use of products**	**Dissipative losses**
*1990*	1,081,463	292,604	87	92,386	55
*1991*	1,038,711	305,212	92	95,733	57
*1992*	987,763	310,328	93	102,213	58
*1993*	976,554	308,049	93	96,751	59
*1994*	957,187	314,585	95	107,276	59
*1995*	954,934	319,378	96	105,464	60
*1996*	974,196	323,224	97	102,637	61
*1997*	945,931	329,652	99	106,317	62
*1998*	936,932	334,201	101	104,468	63
*1999*	908,760	339,103	103	110,308	64
*2000*	911,909	348,033	106	106,915	64
*2001*	927,739	352,304	108	104,128	65
*2002*	910,622	353,001	108	99,746	66
*2003*	911,374	347,719	107	96,253	66
*2004*	896,670	350,019	108	91,965	67
*2005*	875,831	350,271	109	96,933	65
*2006*	887,073	361,380	113	96,132	66
*2007*	860,089	373,110	123	91,065	67
*2008*	862,666	377,110	121	90,570	66
*2009*	796,794	358,738	108	94,703	66
*2010*	841,175	372,168	118	84,515	67
*2011*	821,070	386,690	113	89,347	68
*2012*	825,200	380,576	109	80,748	68
*2013*	843,438	385,729	111	81,677	68
*2014*	802,511	400,953	104	78,983	70
*2015*	799,654	402,229	106	79,215	71

**Table 9:** Components of Japan׳s domestic processed output from 1990 to 2015. All units are in thousand tonnes.

**Japan׳s Domestic Processed Output**					
**Unit: thousand tonnes**					
**Year**	**Emissions to air**	**Emissions to land**	**Emissions to water**	**Dissipative use of products**	**Dissipative losses**
*1990*	1,168,901	445,179	n/a	45,669	n/a
*1991*	1,177,255	448,716	n/a	45,476	n/a
*1992*	1,187,121	453,678	n/a	45,554	n/a
*1993*	1,179,867	447,173	n/a	44,914	n/a
*1994*	1,241,446	455,991	n/a	46,294	n/a
*1995*	1,254,988	446,036	n/a	44,436	n/a
*1996*	1,267,678	455,757	n/a	45,568	n/a
*1997*	1,265,478	466,054	n/a	45,181	n/a
*1998*	1,229,937	460,085	n/a	43,894	n/a
*1999*	1,264,855	451,245	n/a	43,433	n/a
*2000*	1,286,127	460,871	n/a	43,181	n/a
*2001*	1,268,908	452,340	n/a	41,807	n/a
*2002*	1,305,793	444,844	n/a	42,195	n/a
*2003*	1,310,579	463,230	n/a	42,153	n/a
*2004*	1,309,457	470,532	n/a	41,697	n/a
*2005*	1,316,485	474,397	n/a	41,473	n/a
*2006*	1,295,683	470,521	n/a	42,275	n/a
*2007*	1,330,151	470,241	n/a	41,603	n/a
*2008*	1,245,259	451,767	n/a	39,064	n/a
*2009*	1,172,328	435,998	n/a	39,159	n/a
*2010*	1,223,267	431,347	n/a	39,657	n/a
*2011*	1,271,248	426,636	n/a	36,690	n/a
*2012*	1,305,534	424,371	n/a	34,450	n/a
*2013*	1,320,928	429,516	n/a	35,969	n/a
*2014*	1,273,637	437,157	n/a	35,293	n/a
*2015*	1,232,028	433,886	n/a	33,631	n/a

**Table 10:** Components of the United States of America׳s domestic processed output from 1990 to 2015. All units are in thousand tonnes.

**United States of America׳s Domestic Processed Output**					
**Unit: thousand tonnes**					
**Year**	**Emissions to air**	**Emissions to land**	**Emissions to water**	**Dissipative use of products**	**Dissipative losses**
*1990*	5,352,042	439,806	n/a	224,691	n/a
*1991*	5,297,921	439,486	n/a	224,572	n/a
*1992*	5,396,554	455,111	n/a	231,330	n/a
*1993*	5,504,104	467,604	n/a	236,946	n/a
*1994*	5,597,307	486,479	n/a	240,344	n/a
*1995*	5,649,286	500,248	n/a	241,854	n/a
*1996*	5,830,175	519,237	n/a	250,356	n/a
*1997*	5,900,322	542,535	n/a	245,179	n/a
*1998*	5,938,844	566,677	n/a	234,955	n/a
*1999*	6,013,867	593,227	n/a	237,777	n/a
*2000*	6,180,205	617,503	n/a	240,604	n/a
*2001*	6,072,388	623,530	n/a	238,727	n/a
*2002*	6,108,496	634,667	n/a	235,698	n/a
*2003*	6,145,439	652,481	n/a	240,693	n/a
*2004*	6,259,374	677,182	n/a	235,219	n/a
*2005*	6,279,701	699,835	n/a	237,324	n/a
*2006*	6,192,124	718,497	n/a	233,141	n/a
*2007*	6,267,817	729,941	n/a	243,525	n/a
*2008*	6,058,235	739,092	n/a	243,420	n/a
*2009*	5,610,961	704,448	n/a	232,266	n/a
*2010*	5,809,952	705,542	n/a	233,876	n/a
*2011*	5,680,362	703,043	n/a	235,520	n/a
*2012*	5,473,447	711,973	n/a	227,356	n/a
*2013*	5,618,648	705,506	n/a	233,598	n/a
*2014*	5,666,083	717,737	n/a	235,517	n/a
*2015*	5,507,858	718,406	n/a	233,633	n/a

## 2 Experimental design, materials, and methods

To complement and evaluate official statistics on solid waste we developed a model based on domestic material consumption data available in the global material flow database [6]. These data are paired with assumptions on product lifespan, which ends up in a stock and flow model that estimates outflows [10], [11]. The global material flow database covers data from 1970 to 2015, and firstly we attempted to estimate solid waste flows with this data span. While sufficient for most materials, 47 years of data are a short period of time in comparison to building lifespan (especially in western countries). For this reason, we developed a secondary domestic material consumption dataset that starts in 1900, compiled using existing data or projecting flows based on historical GDP data [12], [13], [14]. We then reran the model with this longer dataset and obtained new results that we compared to the first attempt. We named these two sets of results “short run” (47 years of data) and “long run” (115 years of data). The results are shown in Tables 11 and 12, respectively.

**Table 11:** Short run waste potential for year 2015 for five countries and seven material categories. All units in thousand tonnes.

**Waste potential in 2015, short run**					
**Unit: thousand tonnes**					
	**Australia**	**China**	**Germany**	**Japan**	**United States**
*Crop residues*	2283	43,000	1537	621	17,786
*Timber*	2568	71,876	11,202	24,986	45,932
*Glass and chemicals*	16,777	150,989	19,634	14,895	89,018
*Plastics*	3,821	81,174	17,968	36,948	117,480
*Mining waste*	318,571	2,097,779	25,737	65,251	591,472
*Metals*	13,794	224,690	11,969	40,219	47,601
*C&D non-metallic minerals*	5770	1,154,517	49,732	660,955	119,068

**Table 12:** Long run waste potential for year 2015 for five countries and seven material categories. All units in thousand tonnes.

**Waste potential in 2015, long run**					
**Unit: thousand tonnes**					
	**Australia**	**China**	**Germany**	**Japan**	**United States**
*Crop residues*	2283	43,000	1537	621	17,786
*Timber*	4810	74,470	17,113	33,015	170,551
*Glass and chemicals*	16,777	150,989	19,634	14,895	89,018
*Plastics*	3821	81,174	17,968	36,949	117,483
*Mining waste*	318,571	2,097,779	25,737	65,251	591,472
*Metals*	13,823	224,772	11,979	40,319	47,779
*C&D non-metallic minerals*	36,130	1,295,295	211,460	697,888	645,632
