# Supplementary material for: Data on the domestic processed output, balancing items, and solid waste potential for five major world economies
Source: Data Brief. 2018 Dec 26;22:662–75. doi: 10.1016/j.dib.2018.12.072 (PMC6327735; doi:10.1016/j.dib.2018.12.072)
Supplement: Supplementary file 1 — Supplementary material [file mmc1.docx]

Conflict of interest

The authors declare that there is no conflict of interest in regard to this manuscript.
